# A Safety Review and Meta-Analyses of Bevacizumab and Ranibizumab: Off-Label versus Goldstandard

**DOI:** 10.1371/journal.pone.0042701

**Published:** 2012-08-03

**Authors:** Christine Schmucker, Christoph Ehlken, Hansjuergen T. Agostini, Gerd Antes, Gerta Ruecker, Monika Lelgemann, Yoon K. Loke

**Affiliations:** 1 German Cochrane Institute, Institute of Medical Biometry and Medical Informatics, Department of Medical Biometry and Statistics, University Medical Centre Freiburg, Freiburg, Germany; 2 University Eye Hospital, University Medical Centre Freiburg, Freiburg, Germany; 3 Institute of Medical Biometry and Medical Informatics, Department of Medical Biometry and Statistics, University Medical Centre Freiburg, Freiburg, Germany; 4 Medical Advisory Service of the German Health Insurance Funds, Essen, Germany; 5 School of Medicine, University of East Anglia, Norwich, United Kingdom; Medical University Graz, Austria

## Abstract

**Background:**

We set out a systemic review to evaluate whether off-label bevacizumab is as safe as licensed ranibizumab, and whether bevacizumab can be justifiably offered to patients as a treatment for age-related macular degeneration with robust evidence of no differential risk.

**Methods and Findings:**

Medline, Embase and the Cochrane Library were searched with no limitations of language and year of publication. We included RCTs with a minimum follow-up of one year which investigated bevacizumab or ranibizumab in direct comparison or against any other control group (indirect comparison). Direct comparison (3 trials, 1333 patients): The one year data show a significantly higher rate of ocular adverse effects (AE) with bevacizumab compared to ranibizumab (RR = 2.8; 95% CI 1.2–6.5). The proportion of patients with serious infections and gastrointestinal disorders was also higher with bevacizumab than with ranibizumab (RR = 1.3; 95% CI 1.0–1.7). Arterial thromboembolic events were equally distributed among the groups. Indirect comparison: Ranibizumab versus any control (5 trials, 4054 patients): The two year results of three landmark trials showed that while absolute rates of serious ocular AE were low (≤2.1%), relative harm was significantly raised (RR = 3.1; 95% CI 1.1–8.9). A significant increase in nonocular haemorrhage was also observed with ranibizumab (RR = 1.7; 95% CI 1.1–2.7). Bevacizumab versus any control (3 trials, 244 patients): We were unable to judge the safety profile of bevacizumab due to the poor quality of AE monitoring and reporting in the trials.

**Conclusions:**

Evidence from head-to-head trials raises concern about an increased risk of ocular and multiple systemic AE with bevacizumab. Therefore, clinicians and patients should continue to carefully weight up the benefits and harms when choosing between the two treatment options. We also emphasize the need for studies that are powered not just for efficacy, but for defined safety outcomes based on the signals detected in this systematic review.

## Introduction

Age-related macular degeneration (AMD) is the leading cause of irreversible blindness in people over the age of 50 in the developed world [1]. Although an estimated 80% of patients with AMD have the non-neovascular form [2], the neovascular (wet or exudative) form is responsible for almost 90% of severe visual loss resulting from AMD [3].

Anti-angiogenic therapy, e.g., anti-vascular endothelial growth factors (anti-VEGF), which aims to prevent further neovascularization rather than only destroy it, is the latest approach to the treatment of neovascular AMD. Currently, the most commonly used VEGF antagonists are ranibizumab (Lucentis, Genentech, Inc., South San Francisco, CA) and bevacizumab (Avastin; Genentech, Inc., South San Francisco, CA).

Ranibizumab, which is an antibody fragment form the bevacizumab molecule with an increased binding affinity for all forms of VEGF, has been approved for the treatment of patients with neovascular AMD by the Food and Drug Administration and by the European Mediciens Agency since 2006 and 2007, respectively. The costs of ranibizumab, however, are immense. Using monthly injections with a dose of 0.5 mg, the annual costs come to more than US$23 000 per patient [4].

In contrast to ranibizumab, bevacizumab was not developed for the treatment of AMD and consequently has no regulatory approval for this indication or mode of administration. Bevacizumab is approved for the treatment of specific cancers, e.g., metastatic colorectal cancer. In chemotherapy regimens, bevacizumab is associated with an increased risk of thromboembolic events [5], haemorrhage [6] and mortality [7]. However, intravitreal bevacizumab is administered at a dose of 1 to 2.5 mg, which is at least 150 times less than the systemic dose used in chemotherapy [8]. The first report of intravitreal bevacizumab administration for neovascular AMD was published in 2005 [9]. After this initial report, numerous case series which (apparently) support the efficacy and safety of bevacizumab were published [10]–[13]. The costs of intravitreal bevacizumab are much less than for ranibizumab. A single dose of bevacizumab costs 40 times less than a single dose of ranibizumab [4]. This cost differential has important economic implications when extrapolated to the more than 250,000 patients who are treated for neovascular AMD annually in the United States. It is obvious that the low costs and the promising results on visual acuity have led to a widespread off-label use of bevacizumab.

Recently, a long awaited head-to-head comparison from the United States has been published [14]. The results of this trial support the effectiveness of bevacizumab and the authors conclude that both anti-VEGF have equivalent effects on visual acuity when administered according to the same schedule.

However, up to now, safety and tolerability of bevacizumab in comparison to ranibizumab have not been sufficiently assessed. For example, our group conducted a critical assessment of bevacizumab mainly based on the large number of published case series [15]. This previous review highlighted that the perceived low rates of adverse effects for bevacizumab are not supported by reliable data from this study design. Therefore, we performed a systematic review based on randomised controlled clinical trials (RCTs), including latest results of head-to-head comparisons, to address the crucial question whether the available information allow us to judge that unlicensed therapy with bevacizumab is as safe as licensed therapy with ranibizumab, and whether clinicians are justified in offering it to their patients with AMD as a medication with no additional risk. Besides comparing both drugs, we also evaluated whether adverse effects are dose-related.

## Methods

### Search Strategy

We searched Medline, Premedline, Embase and the Cochrane library from inception until May 2011. The search strategy was based on combinations of medical subject headings and keywords and was not restricted to specific languages or years of publication. The search strategy used in Medline is presented in Text S1. Search strategies for other databases were modified to meet the requirements of each database. The literature search also included terms associated with diabetic macular oedema. However, the results of this search will be presented in a separate review. The searches were supplemented by handsearching the bibliographies of included studies and reviews and by contacting the pharmaceutical manufacturer (Genentech) of ranibizumab and bevacizumab. Currently conducted RCTs comparing Avastin® versus Lucentis® were searched both in the register for clinical trials (http://clinicaltrials.gov/) and in the WHO International Clinical Trials Registry Platform (http://www.who.int/ictrp/en/).

### Inclusion Criteria

We included randomised Phase III/IV trials which investigated bevacizumab or ranibizumab in direct comparisons (head-to-head studies) or against any other control group (for potential indirect comparison) in patients with neovascular AMD. RCTs which compared different treatment regimens of ranibizumab or bevacizumab were also included in our systematic review. To address long-term harm, such as myocardial infarction or stroke, one year follow-up data had to be available.

Studies which included patients with other indications than exudative AMD, patients previously treated with VEGF inhibitors or patients receiving systemic anti-VEGF therapy were excluded. We also excluded RCTs which enrolled less than 20 patients.

### Data Extraction and Quality Assessment

Titles and abstracts were reviewed using the above mentioned selection criteria which were also predefined in our study protocol. Full papers of appropriate studies were obtained for detailed evaluation. Data extraction and quality assessment was carried out after a modified evaluation tool of the Center for Reviews and Dissemination (Chapter 4, Systematic Reviews of Adverse Effects) [16]. Information on the number of participants, ascertainment of exposure (e.g., dosage and frequency of drug administered), follow-up time, comparability of groups, definition of expected adverse effects, method used to collect adverse effects data, ascertainment of outcomes (ocular and systemic adverse effects) and transparency of patient flow were abstracted. All stages of study selection, data extraction and quality assessment were done independently by two reviewers (CS and CE). Any disagreement was resolved by discussion and consensus.

### Statistical Analysis

Data from head-to-head studies, studies which compared ranibizumab versus any other treatment than anti-VEGF, and studies which evaluated different dosages of ranibizumab were analysed using the *R* software [17]. This programme was used to compute statistics and generate forest plots to compare safety outcomes of different treatment arms using risk ratios (RR). A chi-square test (p-value<0.05) and an I^2^ test were used to test for statistical heterogeneity between studies. We used the fixed effects model (Mantel-Haeszel method) in the meta-analysis of rare events as it has been shown to be the more appropriate and less biased approach compared to the random effects model [18]. A narrative summary was provided for data that were unsuitable for pooling.

## Results

### Included Studies

**Figure 1:**
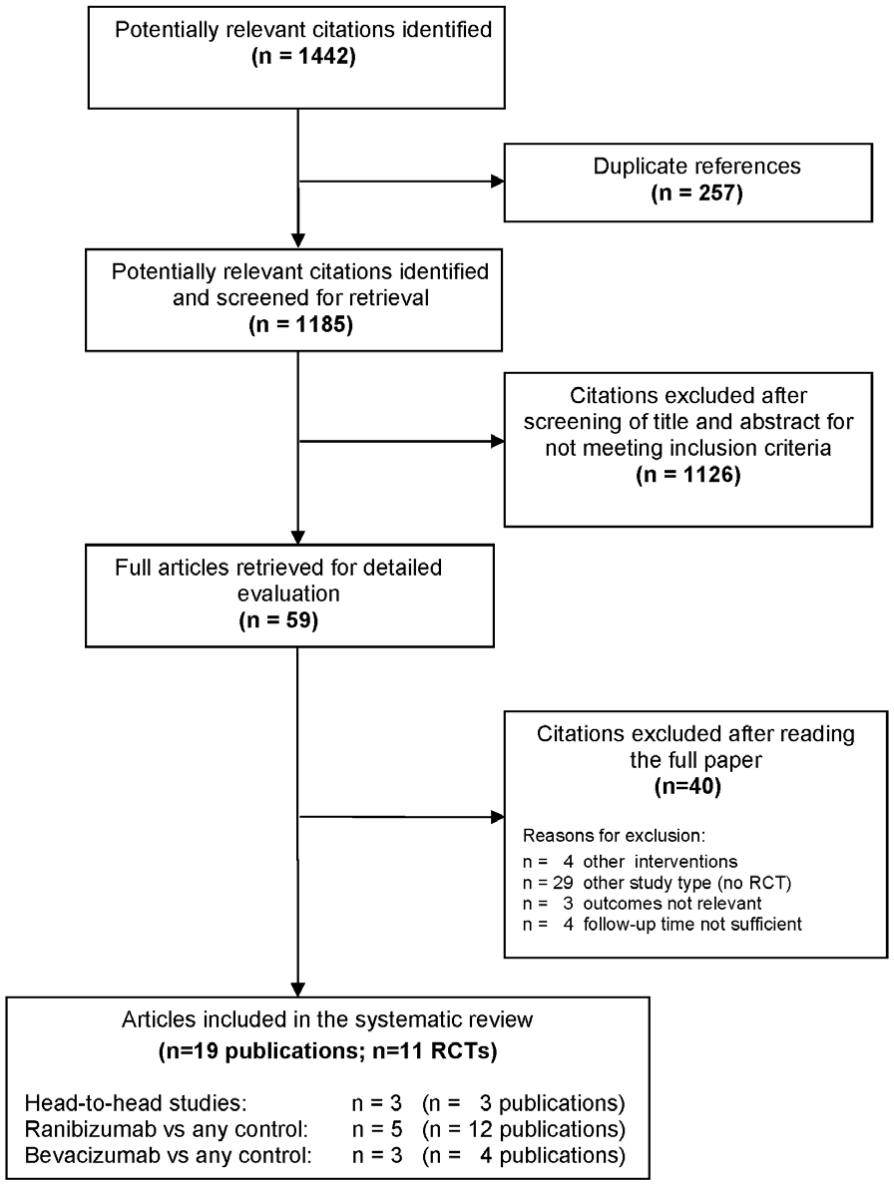
Flow chart of literature search and study selection.

The numbers of studies identified at each stage of the systematic review are shown in Figure 1. After removing duplicate references, the searches identified 1185 citations.

The inclusion criteria were met by 11 RCTs [14], [19]–[28] (19 publications [14], [19]–[36]): three head-to-head studies [14], [19], [20] (three publications) with a total of 1333 patients, five RCTs [21]–[25] (12 publications [21]–[25], [29]–[35]) comparing ranibizumab against any other treatment or dosage with a total of 4054 patients, and three RCTs [26]–[28] (four publications [26]–[28], [36]) comparing bevacizumab against any other treatment with a total of 244 patients.

### Study Characteristics

#### Head-to-head trials

Table 1 shows study characteristic of head-to-head comparisons. The CATT assigned 1208 (1185 finally fulfilled eligibility criteria) patients to receive ranibizumab or bevacizumab on either a monthly schedule or as needed with monthly evaluation [14]. The studies of Biswas et al. [19] and Subramanian et al. [20] were smaller and included 120 and 28 patients, respectively. In these two trials, patients received bevacizumab or ranibizumab monthly for the first three months, followed by a *pro re nata* schedule.

**Table 1:** Characteristics of head-to-head studies comparing ranibizumab with bevacizumab (direct comparison).

	Treated patients				Dosage (mg)		Injections per patient (mean)	
Study	Ranibizumab	Bevacizumab	Follow-up (months)	Treatment regime	Ranibizumab	Bevacizumab	Ranibizumab	Bevacizumab
CATT 2011 [14]	599	586	12	monthly or as needed	0.5	1.25	monthly: 11.7±1.5/as needed: 6.9±3.0	monthly: 11.9±1.2/as needed: 7.7±3.5
Biswas et al. 2011 [19]	60	60	18	monthly for 3 month then as needed	0.5	1.25	5.6	4.3
Subramanian et al. 2010 [20]	8	20	12	monthly for 3 month then as needed	0.5	1.25	4	8

#### Ranibizumab trials for indirect comparison or dose-relationship evaluation

Characteristics of RCTs of ranibizumab are presented in Table 2. The ANCHOR trial compared monthly ranibizumab injections with photodynamic therapy (PDT) and enrolled 423 patients [21]. The MARINA study enrolled 716 patients and compared monthly intravitreal ranibizumab with sham injections [22]. The PIER study also used sham as a comparator and enrolled 184 patients [23]. In contrast to the MARINA study, treated patients received ranibizumab injections once monthly for three consecutive months, followed by a dose administered once every three months. In the SAILOR study, patients were randomised to receive three consecutive monthly injections of 0.3 mg (n = 1169) or 0.5 mg (n = 1209) ranibizumab [24]. After three months, patients were followed by a *pro re nata* schedule. The EXCITE study randomised 353 patients to 0.3 mg quarterly, 0.5 mg quarterly, or 0.3 mg monthly doses of ranibizumab [25]. Treatment comprised a loading phase (three consecutive monthly injections) followed by a nine month maintenance phase (with monthly or quarterly injections).

**Table 2:** Characteristics of RCTs evaluating ranibizumab for indirect comparison.

Study	Included patients	Ranibizumab treated patients	Control treatment	Follow-up (months)	Treatment regime	Dosage (mg)	Injections per patient (mean)
ANCHOR 2009 [21]	423	280	PDT	24	monthly	0.3 and 0.5	24
MARINA 2006 [22]	716	478	sham	24	monthly	0.3 and 0.5	21
PIER 2010 [23]	184	121	sham	24	monthly for 3 month then every 3 month	0.3 and 0.5	nr
SAILOR 2009* [24]	2378	0.3 mg: 1169/0.5 mg: 1209	different dosages of ranibizumab	12	monthly for 3 month then as needed	0.3 and 0.5	4.6 (both groups)
EXCITE 2011 [25]	353	0.3 mg quarterly: 120/0.5 mg quarterly: 118/0.3 mg monthly: 115	different dosages of ranibizumab	12	monthly for 3 month then quarterly vs monthly	0.3 and 0.5	5.7 (0.3 mg quarterly)/5.5 (0.5 mg quarterly)/11.4 (0.3 mg monthly)

Nr: Not reported, PDT: Photodynamic therapy.

* *Cohort I* enrolled randomised patients, therefore, we included this group in the systematic review.

#### Bevacizumab trials for indirect comparison or dose-relationship evaluation

Study characteristics of RCTs comparing bevacizumab with other treatment options are illustrated in Table 3. Sacu et al. assigned 28 patients to bevacizumab or PDT in combination with triamcinolone [26]. Patients received bevacizumab injections once monthly for three consecutive months, followed by a dose administered *pro re nata*. Costagliola et al. enrolled 85 patients and randomised them to bevacizumab as monotherapy or to bevacizumab in combination with PDT [27]. After the first bevacizumab injection retreatment was based on a *pro re nata* schedule. The ABC trial included 131 patients [28]. Bevacizumab was administered once every six weeks. After the first three injections, standardised criteria to decide about retreatment were applied. Patients in the control arm received standard treatment (dependent on the treatment available for different lesion types at the start of the trial).

**Table 3:** Characteristics of RCTs evaluating bevacizumab for indirect comparison.

Study	Included patients	Bevacizumab treated patients	Control treatment	Follow-up (months)	Treatment regime	Dosage (mg)	Injections per patient (mean)
Sacu et al. 2009 [26]	28	14	PDT+T	12	monthly for 3 month then as needed	1.0	6.8
Costagliola et al. 2010 [27]	85	45	PDT+B	12	as needed	1.25	4.6
ABC Trial 2010 [28]	131	65	usual care	12	once every 6 weeks then as needed	1.25	7.1

B: Bevacizumab, PDT: Photodynamic therapy, T: Triamcinolone.

### Ocular Adverse Effects

#### Head-to-head trials

**Figure 2:**
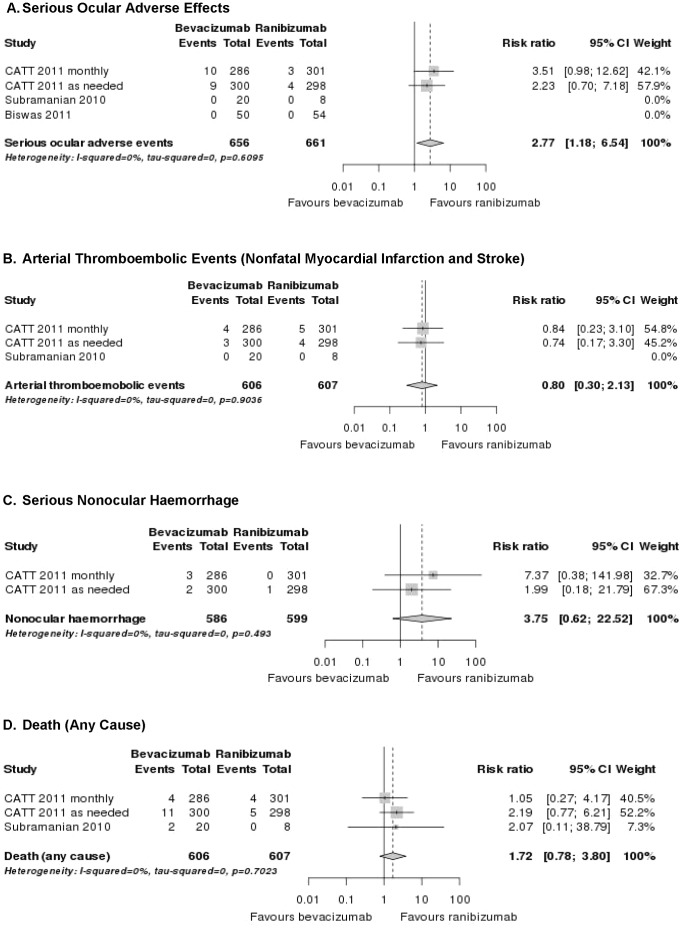
Forest plots: pooled results of head-to-head studies for different safety outcomes.

In the CATT [14], serious ocular adverse effects rates reported with intravitreal anti-VEGF were: endophthalmitis *(ranibizumab: ≤0.7%, bevacizumab: ≤1.4%)*, uveitis *(ranibizumab: ≤0.3%, bevacizumab: ≤0.7%)*, retinal/choroidal detachment *(ranibizumab: 0.0%, bevacizumab: ≤1.0%)*, retinal tear *(ranibizumab: ≤0.3%, bevacizumab: ≤0.3%)*, ocular vessel embolism or occlusion *(ranibizumab: ≤0.7%, bevacizumab: ≤0.7%)* and vitreous haemorrhage *(ranibizumab: ≤0.3%, bevacizumab: ≤0.3%)* both for the monthly and as needed scheme (Table 4). A pooled analysis of serious ocular adverse effects indicated a significantly increased RR for bevacizumab when compared to ranibizumab (RR = 2.8; 95% CI 1.2–6.5; Figure 2a).

**Table 4:** Rates of ocular adverse effects of head-to-head studies (direct comparison).

Study	Endophthalmitis (%)		Uveitis (%)		Retinal/choroidal detachment (%)		Retinal tear (%)		Ocular vessel embolism/occlusion (%)		Vitreous haemorrhage (%)	
	Ranibizumab	Bevacizumab	Ranibizumab	Bevacizumab	Ranibizumab	Bevacizumab	Ranibizumab	Bevacizumab	Ranibizumab	Bevacizumab	Ranibizumab	Bevacizumab
CATT 2011 monthly [14]	0.7	1.4	0.3*	0.3*	0.0	0.7	0.0	0.3	0.0	0.7	0.0	0.0
CATT 2011 as needed [14]	0.0	0.0	0.0*	0.7*	0.0	1.0	0.3	0.3	0.7	0.7	0.3	0.3
Biswas et al. 2011 [19]	nr	nr	nr	nr	nr	nr	nr	nr	nr	nr	nr	nr
Subramanian et al. 2010 [20]	0.0	0.0	0.0	0.0	0.0	0.0	nr	nr	nr	nr	0.0	0.0

Nr: Not reported.

* Percentage refers to uveitis, scleritis, and anterior chamber inflammation.

The two head-to-head studies with comparatively low patient numbers did not indicate major safety concerns: Subramanian et al. [20] reported zero rates for serious ocular adverse effects and Biswas et al. [19] reported only minor complications without specifying them (the rate for ranibizumab was 7.3% and for bevacizumab 11.1%).

#### Ranibizumab trials for indirect comparison

Intravitreal ranibizumab injections have been associated with endophthalmitis (≤2.1%), uveitis (≤1.3%), retinal detachment (≤1.5%), retinal tear (≤1.7%), traumatic lens damage (≤0.9%) and vitreous haemorrhage (≤1.5%) (Table 5) [21], [22], [23], [24], [25].

**Table 5:** Rates of ocular adverse effects of RCTs evaluating ranibizumab for indirect comparison and dose-relationship evaluation.

Study	Endophthalmitis (%)			Uveitis (%)			Retinal detachment (%)			Retinal tear (%)			Lens damage (traumatic) (%)			Vitreous haemorrhage (%)		
	0.3 mg	0.5 mg	PDT	0.3 mg	0.5 mg	PDT	0.3 mg	0.5 mg	PDT	0.3 mg	0.5 mg	PDT	0.3 mg	0.5 mg	PDT	0.3 mg	0.5 mg	PDT
ANCHOR 2009 [21]	**0.0**	**2.1**	**0.0**	**0.0**	**0.7**	**0.0**	**1.5**	**0.0**	**0.7**	**0.0**	**0.7**	**0.0**	**0.0**	**0.0**	**0.0**	**1.5**	**0.0**	**0.0**
	0.3 mg	0.5 mg	Sham	0.3 mg	0.5 mg	Sham	0.3 mg	0.5 mg	Sham	0.3 mg	0.5 mg	Sham	0.3 mg	0.5 mg	Sham	0.3 mg	0.5 mg	Sham
MARINA 2006 [22]	**0.8**	**1.3**	**0.0**	**1.3**	**1.3**	**0.0**	**0.0**	**0.0**	**0.4**	**0.4**	**0.4**	**0.0**	**0.0**	**0.4**	**0.0**	**0.4**	**0.4**	**0.8**
PIER 2010 [23]	**0.0**	**0.0**	**0.0**	**0.0**	**0.0**	**0.0**	**0.0**	**0.0**	**0.0**	**0.0**	**0.0**	**0.0**	**0.0**	**0.0**	**0.0**	**nr**	**nr**	**nr**
	0.3 mg	0.5 mg		0.3 mg	0.5 mg		0.3 mg	0.5 mg		0.3 mg	0.5 mg		0.3 mg	0.5 mg		0.3 mg	0.5 mg	
SAILOR 2009 [24]	**0.2**	**0.4**		**0.1**	**0.2**		**0.1**	**0.0**		**0.0**	**0.1**		**0.1**	**0.1**		**0.3**	**0.1**	
	0.3 mg*	0.5 mg*	0.3 mg#	0.3 mg*	0.5 mg*	0.3 mg#	0.3 mg*	0.5 mg*	0.3 mg#	0.3 mg*	0.5 mg*	0.3 mg#	0.3 mg*	0.5 mg*	0.3 mg#	0.3 mg*	0.5 mg*	0.3 mg#
EXCITE 2011 [25]	**nr**	**nr**	**nr**	**nr**	**nr**	**nr**	**0.8**	**0.0**	**0.0**	**0.0**	**1.7**	**0.0**	**0.8**	**0.0**	**0.9**	**nr**	**nr**	**nr**

Nr: Not reported.

* Quarterly.

# Monthly.

**Figure 3:**
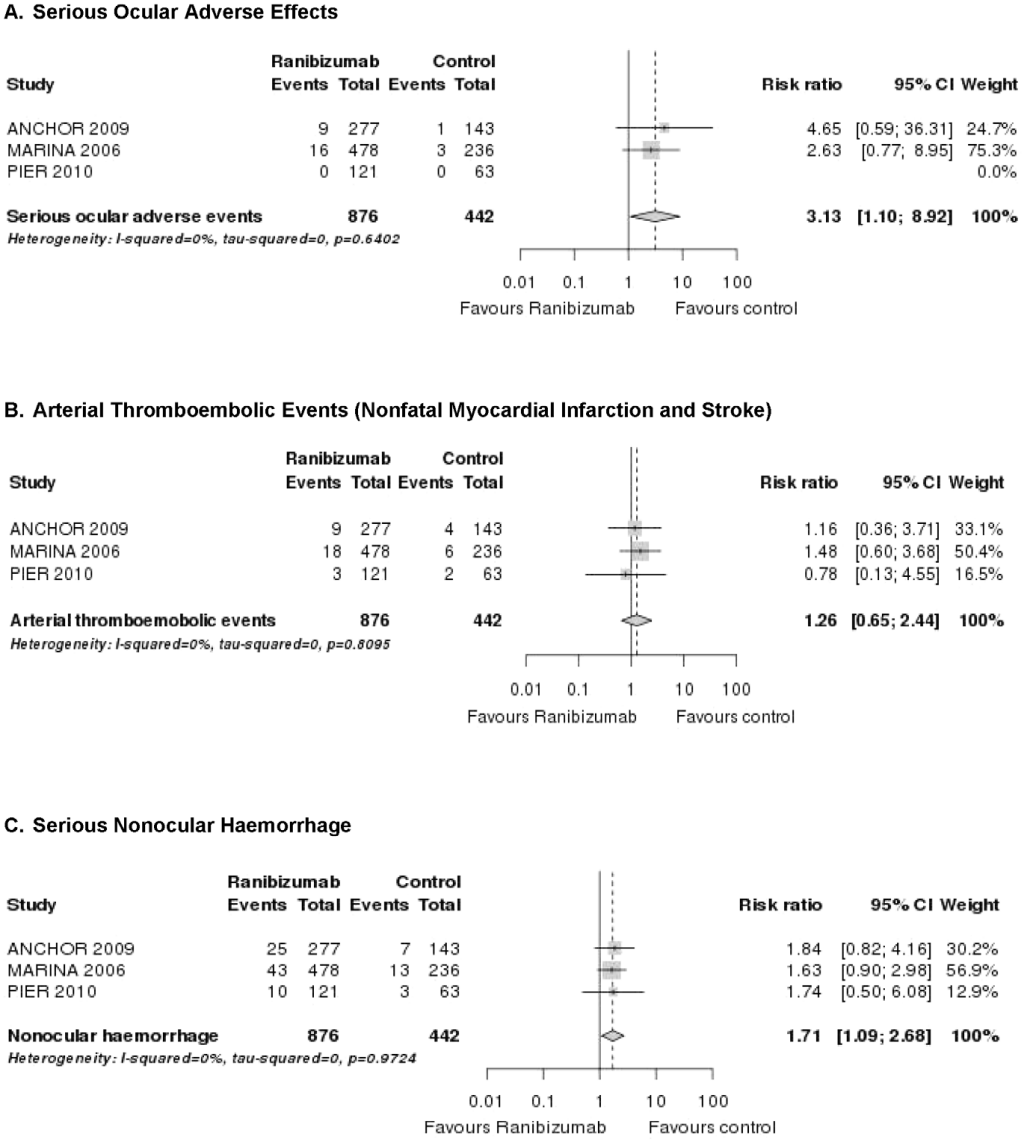
Forest plots: pooled results of RCTs for ranibizumab (any dose vs any control) for different safety outcomes.

A pooled analysis of the ANCHOR [21], MARINA [22] and PIER [23] study showed that while absolute rates of serious ocular adverse effects were low, relative harm was significantly raised compared to controls (RR = 3.1; 95% CI 1.1–8.9; Figure 3a). In addition, these three landmark trials reported a transient increase in intraocular pressure in the study eye after intravitreal injections.

#### Bevacizumab trials for indirect comparison

Serious ocular adverse events associated with bevacizumab were uncommon (Table 6). There were no reported cases of endophthalmitis, retinal detachment, retinal tear and traumatic lens damage. One trial reported a single case of vitreous haemorrhage (2%) and two cases of uveitis (3%) [28].

**Table 6:** Rates of ocular adverse effects of RCTs evaluating bevacizumab for indirect comparison and dose-relationship evaluation.

Study	Endophthalmitis (%)		Uveitis (%)		Retinal detachment (%)		Retinal tear (%)		Lens damage (traumatic) (%)		Vitreous haemorrhage (%)	
	1.0 mg	PDT+T	1.0 mg	PDT+T	1.0 mg	PDT+T	1.0 mg	PDT+T	1.0 mg	PDT+T	1.0 mg	PDT+T
Sacu et al. 2009 [26]	**0.0**	**0.0**	**0.0**	**0.7**	**1.5**	**0.0**	**0.0**	**0.7**	**0.0**	**0.0**	**1.5**	**0.0**
	1.25 mg	PDT+B	1.25 mg	PDT+B	1.25 mg	PDT+B	1.25 mg	PDT+B	1.25 mg	PDT+B	1.25 mg	PDT+B
Costagliola et al. 2010 [27]	**nr**	**nr**	**nr**	**nr**	**nr**	**nr**	**nr**	**nr**	**nr**	**nr**	**nr**	**nr**
	1.25 mg	UC	1.25 mg	UC	1.25 mg	UC	1.25 mg	UC	1.25 mg	UC	1.25 mg	UC
ABC trial 2010 [28]	**0.0**	**0.0**	**3.0**	**2.0**	**0.0**	**2.0**	**0.0**	**0.0**	**0.0**	**0.0**	**2.0**	**0.0**

B: Bevacizumab. Nr: Not reported. T: Triamcinolone. UC: Usual care.

#### Dose-relationship evaluation

**Figure 4:**
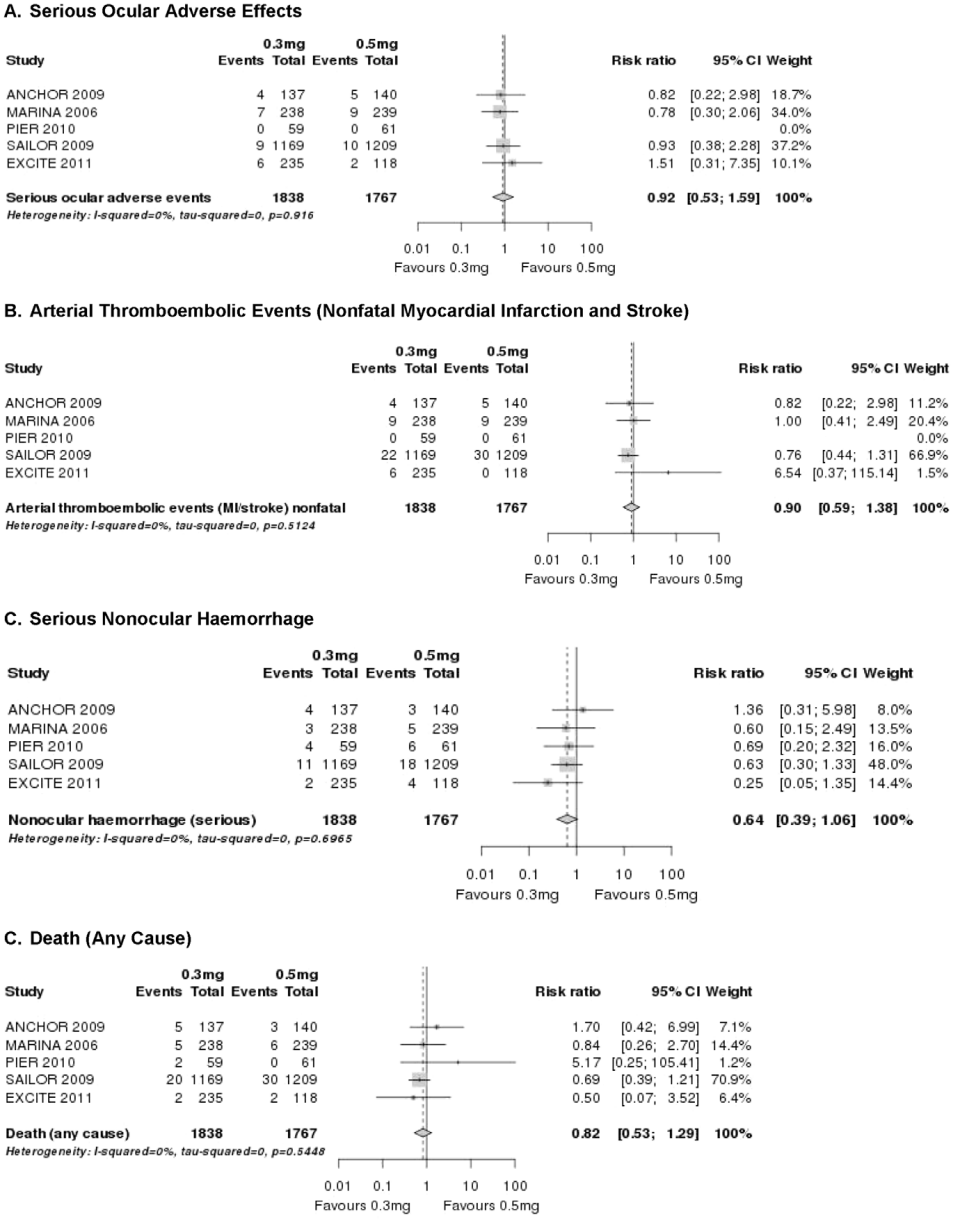
Forest plots: pooled results of ranibizumab 0.3 mg vs 0.5 mg for different safety outcomes.

The incidence of serious ocular adverse effects was low and the magnitude of risk did not appear to be increased with higher doses of ranibizumab as compared to the lower dose (RR = 0.9; 95% CI 0.5–1.6; Figure 4a). We were unable to judge the safety profile for different time frames due to the large variety of applications schemata used in the ranibizumab trials. No safety conclusions can be drawn for the optimal dose-relationship of bevacizumab due to a lack of data.

### Nonocular Adverse Effects

#### Head-to-head trials

In the CATT [14] and in the study of Subramanian et al. [20] more patients died in the bevacizumab than in the ranibizumab group (5.1% *versus* 3.0% and 7.1% *versus* 0.0%. respectively) (Table 7). However, these differences were not statistically significant (RR = 1.7; 95% CI 0.8–3.8; Figure 2d). Nonfatal arterial thromboembolic events (myocardial infarction and stroke) were similar distributed among the treatments groups (approximately 1%; RR = 0.8; 95% CI 0.3–2.1; Figure 2b). In contrast, the proportion of patients with serious systemic adverse effects (primarily hospitalisations due to infections such as pneumonia or urinary tract infections and gastrointestinal disorders such as haemorrhage, nausea and vomiting) was significantly higher with bevacizumab than with ranibizumab (RR = 1.3; 95% CI 1.0–1.7; cumulative data from CATT publication). The rate of serious nonocular haemorrhage (duodenal ulcer haemorrhage, gastric ulcer haemorrhage, lower gastrointestinal haemorrhage and rectal haemorrhage) was numerically, but not statistically significantly higher in the bevacizumab than in the ranibizumab arm (1.0 and 0.7% *versus* 0.0 and 0.3%, respectively [Table 7]). A pooled analysis indicated that there may be a safety signal (RR = 3.8; 95% CI 0.6–22.5; Figure 2c). Biswas et al. did not mention systemic adverse effects in their head-to-head comparison [19].

**Table 7:** Rates of systemic adverse effects of head-to-head studies (direct comparison).

Study	Death (any cause) (%)		Myocardial infarction (%)		Cerebrovascular accident (%)		Nonocular haemorrhage (%)		Infections (%)	
	Ranibizumab	Bevacizumab	Ranibizumab	Bevacizumab	Ranibizumab	Bevacizumab	Ranibizumab	Bevacizumab	Ranibizumab	Bevacizumab
CATT 2011 monthly [14]	1.3	1.4	0.7	0.7	1.0	0.7	0.0*	1.0*	2.0#	3.8#
CATT 2011 as needed [14]	1.7	3.7	1.0	0.3	0.3	0.7	0.3*	0.7*	4.0#	6.0#
Biswas et al. 2011 [19]	nr	nr	nr	nr	nr	nr	nr	nr	nr	nr
Subramanian et al. 2010 [20]	0.0	7.1**	0.0	0.0	0.0	0.0	nr	nr	nr	nr

Nr: Not reported.

* Data from CATT appendix: they refer to duodenal ulcer haemorrhage, gastric ulcer haemorrhage, (lower) gastrointestinal haemorrhage, rectal haemorrhage.

# Rates refer mainly to pneumonia and urinary tract infections, other infections are not clearly specified.

** Two patients died in the Bevacizumab group (meckel cell carcinoma and unknown cause).

#### Ranibizumab trials for indirect comparison

The rates of nonocular serious adverse effects of single RCTs are displayed in Table 8. The rate of key arterial nonfatal thromboembolic effects (myocardial infarction and stroke) during the first and second year of the ANCHOR [21] and MARINA [22] trials was numerically, but not statistically significantly higher in the 0.5 mg arm than in the control arm (3.6% [21] and 2.5% [22], respectively *versus* 1.4% and 0.8%, respectively). In the ANCHOR [21], MARINA [22] and PIER [23] study, the incidence of serious nonocular haemorrhage (such as gastrointestinal haemorrhage, traumatic subdural haematoma and duodenal ulcer haemorrhage) was also consistently higher in the ranibizumab than in the control groups (2.9% [0.3 mg] [21], 2.1% [0.5 mg] [22] and 9.8% [0.5 mg] [23] *versus* 0.7%, 0.8% and 4.8%). A pooled analysis indicated that this risk reached the standard thresholds for statistical significance (RR = 1.7; 95% CI 1.1–2.7; Figure 3c). Except for the EXCITE study [25], increased infection rates were not reported in the ranibizumab trials.

**Table 8:** Rates of systemic adverse effects of RCTs evaluating ranibizumab for indirect comparison and dose-relationship evaluation.

Study	Death (any cause) (%)			Myocardial infarction (%)			Cerebrovascular accident (%)			Nonocular haemorrhage (%)			Infections (%)		
	0.3 mg	0.5 mg	PDT	0.3 mg	0.5 mg	PDT	0.3 mg	0.5 mg	PDT	0.3 mg	0.5 mg	PDT	0.3 mg	0.5 mg	PDT
ANCHOR 2009 [21]	**3.7**	**2.1**	**3.5**	**0.7**	**3.6**	**1.4**	**2.2**	**0.0**	**1.4**	**2.9**	**2.1**	**0.7**	**nr**	**nr**	**nr**
	0.3 mg	0.5 mg	Sham	0.3 mg	0.5 mg	Sham	0.3 mg	0.5 mg	Sham	0.3 mg	0.5 mg	Sham	0.3 mg	0.5 mg	Sham
MARINA 2006 [22]	**2.1**	**2.5**	**2.5**	**2.5**	**1.3**	**1.7**	**1.3**	**2.5**	**0.8**	**1.3**	**2.1**	**0.8**	**nr**	**nr**	**nr**
PIER 2010 [23]	**3.4**	**0.0**	**1.6**	**0.0**	**0.0**	**1.6**	**0.0**	**0.0**	**0.0**	**6.8**	**9.8**	**4.8**	**nr**	**nr**	**nr**
	0.3 mg	0.5 mg		0.3 mg	0.5 mg		0.3 mg	0.5 mg		0.3 mg	0.5 mg		0.3 mg	0.5 mg	
SAILOR 2009 [24]	**1.7**	**2.4**		**1.2**	**1.2**		**0.7**	**1.2**		**0.9**	**1.5**		**nr**	**nr**	
	0.3 mg*	0.5 mg*	0.3 mg#	0.3 mg*	0.5 mg*	0.3 mg#	0.3 mg*	0.5 mg*	0.3 mg#	0.3 mg*	0.5 mg*	0.3 mg#	0.3 mg*	0.5 mg*	0.3 mg#
EXCITE 2011 [25]	**0.0**	**1.6**	**0.9**	**0.8**	**0.0**	**0.9**	**0.8**	**0.0**	**0.9**	**0.0**	**3.4**	**0.9**	**2.5**	**4.2**	**3.5**

Nr: Not reported.

* Quarterly.

# Monthly.

#### Bevacizumab trials for indirect comparison

Different to the ranibizumab trials, intravitreal bevacizumab injections were apparently not associated with an increased risk of nonocular haemorrhage (Table 9). However, this assumption is based on limited details concerning the harms reported within the articles: Two trials mentioned generically that no systemic effects were observed [26], [27]; and one study reported zero rates for nonocular haemorrhage [28]. Taken together, one bevacizumab trial described a single case (2%) of death and one patient (2%) who experienced a myocardial infarction after intravitreal bevacizumab [28].

**Table 9:** Rates of systemic adverse effects of RCTs evaluating bevacizumab for indirect comparison and dose-relationship evaluation.

Study	Death (any cause) (%)		Myocardial infarction (%)		Cerebrovascular accident (%)		Nonocular haemorrhage (%)		Infections (%)	
	1.0 mg	PDT+T	1.0 mg	PDT+T	1.0 mg	PDT+T	1.0 mg	PDT+T	1.0 mg	PDT+T
Sacu et al. 2009 [26]	**nr**	**nr**	**nr**	**nr**	**nr**	**nr**	**nr**	**nr**	**nr**	**nr**
	1.25 mg	PDT+B	1.25 mg	PDT+B	1.25 mg	PDT+B	1.25 mg	PDT+B	1.25 mg	PDT+B
Costagliola et al. 2010 [27]	**nr**	**nr**	**0.0**	**0.0**	**0.0**	**0.0**	**nr**	**nr**	**nr**	**nr**
	1.25 mg	UC	1.25 mg	UC	1.25 mg	UC	1.25 mg	UC	1.25 mg	UC
ABC trial 2010 [28]	**2.0**	**0.0**	**2.0**	**0.0**	**0.0**	**0.0**	**0.0**	**1.0**	**nr**	**nr**

B: Bevacizumab. Nr: Not reported. T: Triamcinolone. UC: Usual care.

#### Dose-relationship evaluation

The rates of key arterial thromboembolic events were similar across dose groups (RR = 0.9; 95% CI 0.6–1.4; Figure 4b). The rates of nonocular haemorrhage, however, showed a difference between doses, with higher rates in the 0.5 mg dose group compared with the 0.3 mg dose group (Table 8). The total number of events was comparatively small, and the difference was not fully confirmed statistically (RR = 0.6; 95% CI 0.4–1.1; Figure 4c). The incidence of death is not dose related (RR = 0.8; 95% CI 0.5–1.3; Figure 4d). We could not evaluate whether there is a difference in safety outcomes in a less than monthly regimen for ranibizumab due to study heterogeneity. Again, no safety conclusions regarding optimal doses of intravitreal bevacizumab can be drawn due to a lack of data.

### Summary of Methodological Quality and Risk of Bias

#### Head-to-head trials

The methodological quality of the head-to-head studies is presented in Table 10. In one trial patients and investigators were adequately blinded [20]. However, a small sample size, an almost male population and a lack of any description as to how adverse effects were rigorously monitored, as well as the inadequate reporting of actual events does not allow a reliable conclusion on safety outcomes. Similar to Subramanian et al. [20], Biswas et al. [19] also showed large deficiencies in their study methodology. Therefore, no reliable conclusions on safety can be drawn on the basis of these two studies.

**Table 10:** Methodological quality of head-to-head studies comparing ranibizumab with bevacizumab (direct comparison).

Study	Comparability of groups	Adequate blinding	Definition of expected AE	Definition of method used to collect AE data	Transparency of patient flow	Validity safety
CATT 2011 [14]	yes*	single blind#	yes	yes	unclear	moderate- high
Biswas et al. 2011 [19]	not specified	single blind**	no	no	unclear	low
Subramanian et al. 2010 [20]	no	double blind	in part	no	unclear	low

AE: Adverse effects.

* The CATT showed only minor differences in the socioeconomic status and in the history of myocardial infarction between the randomised groups.

# Outcome assessor and care provider blinded, patient initially masked, billing statement may unmask.

** All assessors were masked. Unclear whether patients were masked.

The CATT showed no substantial imbalances in the demographic or ocular characteristics of the study groups at baseline [14]. Adverse effects were, in contrast to the two other head-to-head trials, rigorously monitored and adequately reported. Due to the billing status, masking of patients could not be maintained. The adjudication of serious adverse effects could, however, most likely be secured by a medical monitor who reviewed serious adverse effects and was unaware of study group assignment.

It was outstanding that in none of the three head-to-head trials reasons for drop-outs were given. In addition, flow charts documenting the patient flow were missing.

#### Ranibizumab trials for indirect comparison or dose-relationship evaluation

Three of the ranibizumab trials were of high methodological quality (comparability of groups, adequate blinding, high patient numbers, transparency of patient flow, definition of expected adverse effects and method used to collect adverse effects data; Table 11) [21]–[23]. The remaining two studies (SAILOR [24] and EXCITE [25]) showed deficiencies in the definition and method used to collect expected adverse effects data.

**Table 11:** Methodological quality of RCTs evaluating ranibizumab for indirect comparison.

Study	Comparability of groups	Adequate blinding	Definition of expected AE	Definition of method used to collect AE data	Transparency of patient flow	Validity safety
ANCHOR 2009 [21]	yes	double blind	yes	yes	yes	high
MARINA 2006 [22]	yes	double blind	yes	yes	yes	high
PIER 2008 [23]	yes	double blind	yes	yes	yes	high
SAILOR 2009 [24]	yes	single (patient)	in part	no	yes	moderate
EXCITE 2011 [25]	in part	double blind	in part	no	yes*	moderate/low

AE: Adverse effects.

* It was outstanding that in the 0.5 mg group 10.2% of patients discontinued because of adverse effects, in the 0.3 mg quarterly group 3.3% and in the 0.3 mg monthly group 4.3%, respectively.

#### Bevacizumab trials for indirect comparison or dose-relationship evaluation

Except for the ABC trial [28] the results of RCTs evaluating bevacizumab are of limited values (Table 12). The main limitations stemmed from the lack of blinding and the lack of any description as to how adverse effects were monitored, as well as the inadequate reporting of actual events. In addition to these shortcomings, the overall sample size of bevacizumab treated patients was much lower than for ranibizumab treated patients (244 *versus* 4054 patients).

**Table 12:** Methodological quality of RCTs evaluating bevacizumab for indirect comparison.

Study	Comparability of groups	Adequate blinding	Definition of expected AE	Definition of method used to collect AE data	Transparency of patient flow	Validity safety
Sacu et al. 2009 [26]	in part	open label	no	no	yes	low
Costagliola et al. 2010 [27]	not specified	not specified	no	no	unclear	low
ABC Trial 2010 [28]	in part	double blind	in part	in part	yes	moderate

AE: Adverse effects.

## Discussion

### Principal Findings

#### Head-to-head trials

The study results of head-to-head trials show that the rates of serious ocular adverse effects are low (<1.5%), but they indicate a potential safety risk related to the injection procedure under bevacizumab. Because both ranibizumab and bevacizumab are administered intravitreally and the number of received injections did not differ significantly, the higher risk of ocular adverse effects is either the result of a true difference between the drugs or the method of manufacture. It is obvious that using an unlicensed drug is less safe than using a licensed one where the regulatory authority monitors quality control of the manufacturer. Therefore, it is likely that the higher rates of ocular adverse effects of bevacizumab could be the result of the compounding procedures used to prepare the syringes containing bevacizumab. Although the rates for ocular safety outcomes were low, it has to be kept in mind that the cumulative risk will increase with repeated injections, i.e., for every new decision, the same risks have to be taken into account.

The pooled relative risk of CATT also indicates a significant signal of a higher hospitalisation rate due to sepsis, pneumonia or gastrointestinal disorders and a possible signal of an increased risk of nonocular haemorrhage following the intravitreal use of bevacizumab [14]. Arterial thromboembolic events and death were, however, not associated with the use of bevacizumab in AMD. Since information on drop outs were missing - a complete follow-up is, however, necessary to determine if those patients who withdrew due to adverse effects are different from those who did not adhere - no final conclusion can be drawn regarding whether these findings were drug-related or due to chance alone. We also cannot exclude the possibility of measured and unmeasured confounders in the CATT that may have influenced the results. There were some minor differences (such as the socioeconomic status and the history of myocardial infarction) in baseline characteristics between the randomised groups but it would be impossible to accurately predict the direction or magnitude of impact that these differences would have on the results. Sepsis, infections, gastrointestinal disorders and haemorrhage are listed as common serious adverse events (≥2% difference between the trial arms in at least one clinical trial) for bevacizumab, therefore, the pattern observed in CATT may not be entirely atypical (http://www.medicines.org.uk/emc/medicine/15748/SPC/). Equally, the potential lack of blinding in CATT may mean that patients and clinicians who were concerned about these recognised events ended up reporting it more frequently with bevacizumab than with ranibizumab.

#### Ranibizumab and bevacizumab trials for indirect comparison

Our analysis based on three landmark ranibizumab trials indicates a significant increase in nonocular haemorrhage and a significantly higher rate of serious ocular adverse effects under ranibizumab [21]–[23]. The higher risk of endophthalmitis, retinal detachment/tear and vitreous haemorrhage are not surprising in these trials which used PDT or sham as comparator, because these events are attributable to the injection procedure. Overall, most of the RCTs evaluating ranibizumab fulfil the criteria of reporting adverse effects, but very rare adverse effects, i.e., adverse effects with an incidence rate of less than one in 1000, could not be evaluated because the number of patients was still too small.

In contrast to the RCTs evaluating ranibizumab, the trials evaluating bevacizumab showed methodological limitations (e.g., small sample sizes and inadequate reporting of adverse effects). In addition, generally investigators of RCTs tend to select patients who are fitter, healthier and have lower risks than real-life patients. These factors can lead to an underestimation of adverse effects - especially if we also take into account that higher evidence from phase III/IV ranibizumab trials suggests signals for an increased ocular and systemic vascular and haemorrhagic risk and intravenous bevacizumab for the management of cancer is associated with major systemic adverse effects like thromboembolic events and haemorrhage [5], [6]. On the other hand, the risk for developing systemic adverse effects may be much lower in AMD patients who receive a dose of intravitreal bevacizumab that is about 0.25% of that used for intravenous treatment [37], [38].

#### Ranibizumab and bevacizumab trials for dose-relationship evaluation

The rates of safety events between 0.5 mg and 0.3 mg ranibizumab were low and did not suggest that the higher dose has a higher risk of ocular adverse effects, arterial thromboembolic events and death. However, there may be a higher rate of nonocular haemorrhage associated with the 0.5 mg dose. The total number of events was small, and the difference was not confirmed fully statistically, but this finding should be monitored via postmarketing surveillance and ongoing trials. Because the 0.5 mg doses of ranibizumab tend to have a slightly greater visual acuity benefit than 0.3 mg doses in patients with neovascular AMD [21], [22], [24], the decision on how much ranibizumab to use must be decided carefully by the clinician and patient based on the benefit and harm ratio.

### Strengths and Limitations

We did not include non-RCTs in this systematic review. The reason behind is that a previous review of our group evaluated safety for ranibizumab and bevacizumab on the basis of observational studies, mainly case reports [15]. However, in the case of bevacizumab follow-up times are too short, sample sizes too small and the monitoring and reporting of adverse outcomes shows large deficiencies, therefore, no reliable conclusions on safety could be drawn using this study design.

We believe that the crucial question whether adverse effects differ between off-label bevacizumab and licensed ranibizumab can only be answered on the basis of head-to-head trials or RCTs for indirect comparison with reasonable follow-up times and sample sizes. We are aware that data from RCTs could underestimate adverse effects mainly due to the inclusion of highly selected (non-representative) patients and/or publication bias [39]. In addition, small sample sizes limit the ability to detect rare but serious adverse effects [39]. Therefore, it is likely that the results of this review may have resulted in a lower risk of adverse effects than the true risk.

### Other Reviews

A report from the US Food and Drug Administration for intravitreal ranibizumab concluded that there may be a theoretical risk of arterial thromboembolic events [40]. This finding is similar to the result of our meta-analysis of three phase III/IV ranibizumab studies which also shows a possible signal with regard to thromboembolic events (RR = 1.3; 95% CI 0.7–2.4).

Another recent retrospective analysis of 146 942 Medicare case records addressed systemic complications under intravitreal anti-VEGF treatment [41]. Curtis and associates reported higher risks of stroke and all-cause mortality with intravitreal injections of bevacizumab as compared to ranibizumab for the treatment of AMD. Further analysis of the Medicare claims database presented at the 2011 Association for Research in Vision and Ophthalmology (ARVO) annual meeting (Gower EW et al. ARVO 2011 E-Abstract 6644) indicated an 11% higher risk in all-cause mortality and 57% higher risk of haemorrhagic stroke with bevacizumab, with no statistically significant differences in the risk of either myocardial infarction or ischemic stroke. The significance of the results consisted even after adjusting for potential differences in socioeconomic status of the patients.

Van der Reis et al. systematically assessed and compared the incidences of adverse effects of ranibizumab, bevacizumab and pegaptanib [42]. They reported cumulative incidence rates in their review, therefore, we were not able to compare our results with this review directly. However, different to our findings, they summarised that there is no sufficient evidence to conclude that there is a difference between the safety profile of different VEGF inhibitors. The Royal College of Ophthalmologists UK also stated that both drugs have a similar safety profile [43]. This finding is also in contrast to our thorough examinations, which suggests that there remain issues of concern using off-label bevacizumab.

### Implications for Clinical Practice

Despite the completion of the one year CATT results, controversies remain regarding the safety profile of bevacizumab. It is unclear whether the observed differences in serious adverse effects between bevacizumab and ranibizumab are due to genuine differences in systemic toxicity, or whether the data has been affected by possible confounding. We conclude that currently it is not possible to rule out a clinically relevant risk for serious adverse effects under the use of unlicensed bevacizumab. The results from the second year of CATT and from other ongoing multicentre comparative clinical trials in Europe (e.g., the IVAN study in Great Britain, the LUCAS study in Norway, the GEFAL study in France, the MANTA study in Austria, or the VIBERA study in Germany) should help to clarify whether these increased risks of adverse effects are related to intravitreal anti-VEGF therapy. If these signals regarding higher rates of adverse effects are subsequently confirmed to be higher in bevacizumab than in ranibizumab, some of the cost savings with bevacizumab may be negated.

In the meantime, clinicians and patients should continue to carefully weight up the benefits and harms when choosing between the two available treatment options. We also emphasize the need for heightened surveillance for systemic adverse effects with intraocular anti-VEGF injections for AMD and other retinal diseases and studies that are powered not just for efficacy, but for defined safety outcomes based on the signals detected in this systematic review.

## Supporting Information

Text S1 **Search strategy in Medline (Ovid).** Note: The literature search also included terms associated with diabetic macular oedema. However, the results of this search will be presented in a separate review.
(PDF)
