# Supplementary material for: A Safety Review and Meta-Analyses of Bevacizumab and Ranibizumab: Off-Label versus Goldstandard
Source: PLoS One. 2012 Aug 3;7(8):e42701. doi: 10.1371/journal.pone.0042701 (PMC3411814; doi:10.1371/journal.pone.0042701)
Supplement: Text S1 — Search strategy in Medline (Ovid). Note: The literature search also included terms associated with diabetic macular oedema. However, the results of this search will be presented in a separate review. (PDF) [file pone.0042701.s001.pdf]

Text S1 Search strategy in Medline (Ovid).

1. exp Randomized Controlled Trial/
2. exp Controlled Clinical Trial/
3. (randomized or randomised).ab.
4. placebo.ab.
5. clinical trials as topic.sh.
6. randomly.ab.
7. trial.ti.
8. exp Random Allocation/
9. 1 or 2 or 3 or 4 or 5 or 6 or 7 or 8
10. exp animals/ not humans.sh.
11. 9 not 10
12. exp Macular Degeneration/
13. exp Retinal Degeneration/
14. exp Retinal Neovascularization/
15. exp Choroidal Neovascularization/
16. exp Macula Lutea/
17. ((macul* or retina* or choroid*) adj5 degener*).mp. [mp=protocol supplementary concept, rare disease supplementary concept, title, original title, abstract, name of substance word, subject heading word, unique identifier]
18. ((macul* or retina* or choroid*) adj5 neovasc*).mp. [mp=protocol supplementary concept, rare disease supplementary concept, title, original title, abstract, name of substance word, subject heading word, unique identifier]
19. ((macul* or retina* or choroid*) adj5 neo-vasc*).mp. [mp=protocol supplementary concept, rare disease supplementary concept, title, original title, abstract, name of substance word, subject heading word, unique identifier]
20. amd.mp. [mp=protocol supplementary concept, rare disease supplementary concept, title, original title, abstract, name of substance word, subject heading word, unique identifier]
21. cnv.mp. [mp=protocol supplementary concept, rare disease supplementary concept, title, original title, abstract, name of substance word, subject heading word, unique identifier]
22. (subfoveal neovasc* or subfoveal neo-vasc*).mp. [mp=protocol supplementary concept, rare disease supplementary concept, title, original title, abstract, name of substance word, subject heading word, unique identifier]

23. (extrafoveal neovasc* or extrafoveal neo-vasc*).mp. [mp=protocol supplementary concept, rare disease supplementary concept, title, original title, abstract, name of substance word, subject heading word, unique identifier]

24. (juxtafoveal neovasc* or juxtafoveal neo-vasc*).mp. [mp=protocol supplementary concept, rare disease supplementary concept, title, original title, abstract, name of substance word, subject heading word, unique identifier]

25. (occult neovasc* or occult neo-vasc*).mp. [mp=protocol supplementary concept, rare disease supplementary concept, title, original title, abstract, name of substance word, subject heading word, unique identifier]

26. (classic neovasc* or classic neo-vasc*).mp. [mp=protocol supplementary concept, rare disease supplementary concept, title, original title, abstract, name of substance word, subject heading word, unique identifier]

27. (chor* neovasc* or chor* neo-vasc*).mp. [mp=protocol supplementary concept, rare disease supplementary concept, title, original title, abstract, name of substance word, subject heading word, unique identifier]

28. 12 or 13 or 14 or 15 or 16 or 17 or 18 or 19 or 20 or 21 or 22 or 23 or 24 or 25 or 26 or 27

29. exp Diabetic Retinopathy/

30. exp Macular Edema/

31. exp Macular Degeneration/

32. (macular edema cystoid or macular oedema cystoid).mp. [mp=protocol supplementary concept, rare disease supplementary concept, title, original title, abstract, name of substance word, subject heading word, unique identifier]

33. (macula* adj2 edema).mp. [mp=protocol supplementary concept, rare disease supplementary concept, title, original title, abstract, name of substance word, subject heading word, unique identifier]

34. (macula* adj2 oedema).mp. [mp=protocol supplementary concept, rare disease supplementary concept, title, original title, abstract, name of substance word, subject heading word, unique identifier]

35. (DME or DMO).mp. [mp=protocol supplementary concept, rare disease supplementary concept, title, original title, abstract, name of substance word, subject heading word, unique identifier]

36. (CME or CSME).mp. [mp=protocol supplementary concept, rare disease supplementary concept, title, original title, abstract, name of substance word, subject heading word, unique identifier]

37. (CMO or CSMO).mp. [mp=protocol supplementary concept, rare disease supplementary concept, title, original title, abstract, name of substance word, subject heading word, unique identifier]

38. 29 or 30 or 31 or 32 or 33 or 34 or 35 or 36 or 37

39. exp Antibodies, Monoclonal/

40. monoclonal antibod*.mp.

41. exp Angiogenesis Inhibitors/

42. angiogenesis inhibitor*.mp.

43. exp Angiogenesis Inducing Agents/

44. angiogenesis inducing agent*.mp.

45. exp Endothelial Growth Factors/

46. endothelial growth factor*.mp.

47. exp Vascular Endothelial Growth Factor A/

48. vascular endothelial growth factor*.mp.

49. (vegf or (anti adj2 vegf)).mp. [mp=protocol supplementary concept, rare disease supplementary concept, title, original title, abstract, name of substance word, subject heading word, unique identifier]

50. endo-GF.mp.

51. (bevacizumab or avastin).mp. [mp=protocol supplementary concept, rare disease supplementary concept, title, original title, abstract, name of substance word, subject heading word, unique identifier]

52. (ranibizumab or lucentis or rhufab).mp. [mp=protocol supplementary concept, rare disease supplementary concept, title, original title, abstract, name of substance word, subject heading word, unique identifier]

53. 39 or 40 or 41 or 42 or 43 or 44 or 45 or 46 or 47 or 48 or 49 or 50 or 51 or 52

54. 11 and 28 and 53

55. 11 and 38 and 53

56. 54 or 55

57. remove duplicates from 56
